# Supplementary material for: Unraveling the causal association between leukocyte telomere length and infertility: A two-sample Mendelian randomization study
Source: PLoS One. 2024 Mar 21;19(3):e0298997. doi: 10.1371/journal.pone.0298997 (PMC10956861; doi:10.1371/journal.pone.0298997)
Supplement: S1 Fig — (PDF) [file pone.0298997.s001.pdf]

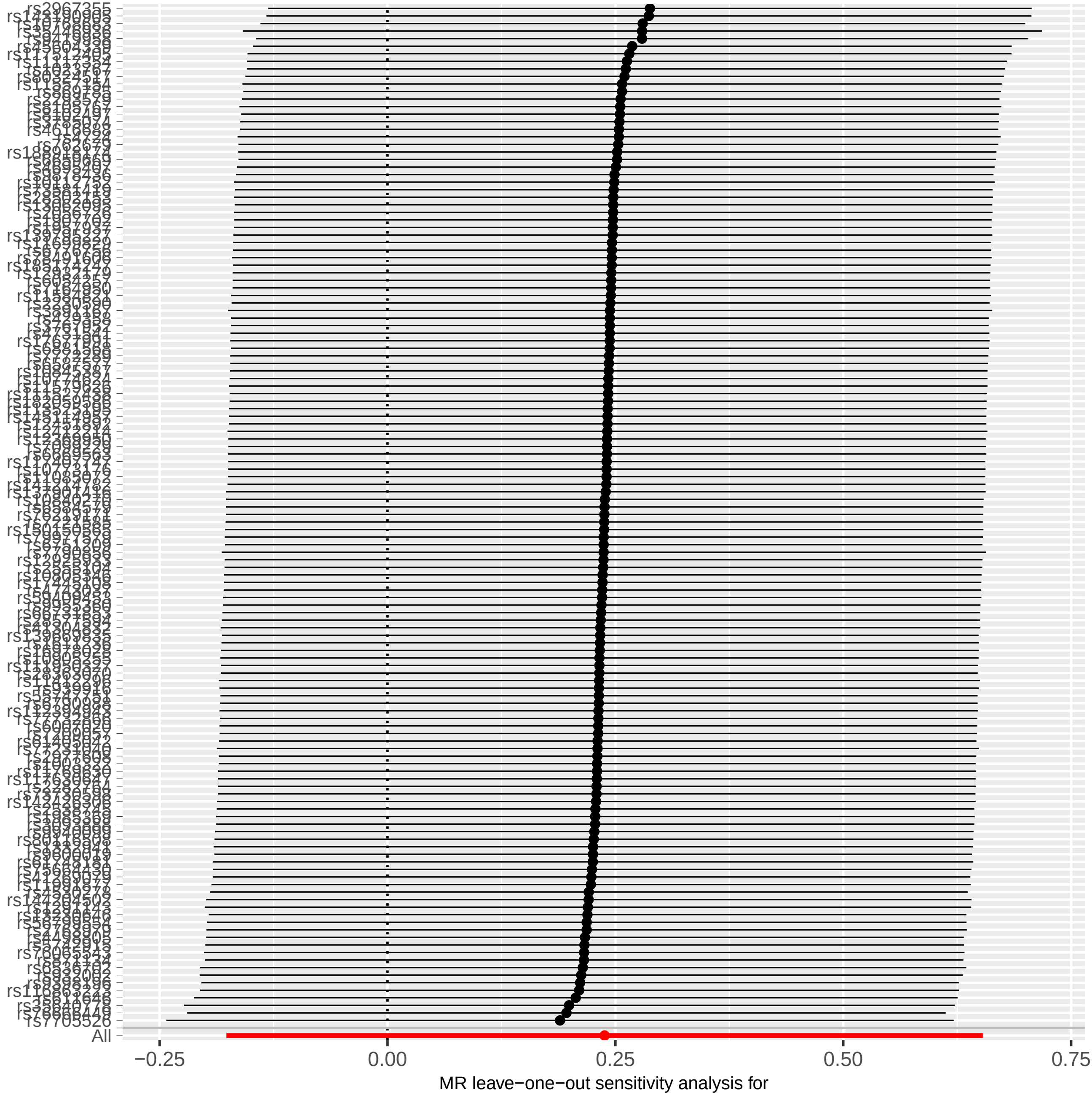

MR leave−one−out sensitivity analysis for
'telomere length || id:ieu−b−4879' on 'Male infertility || id:finn−b−N14_MALEINFERT'

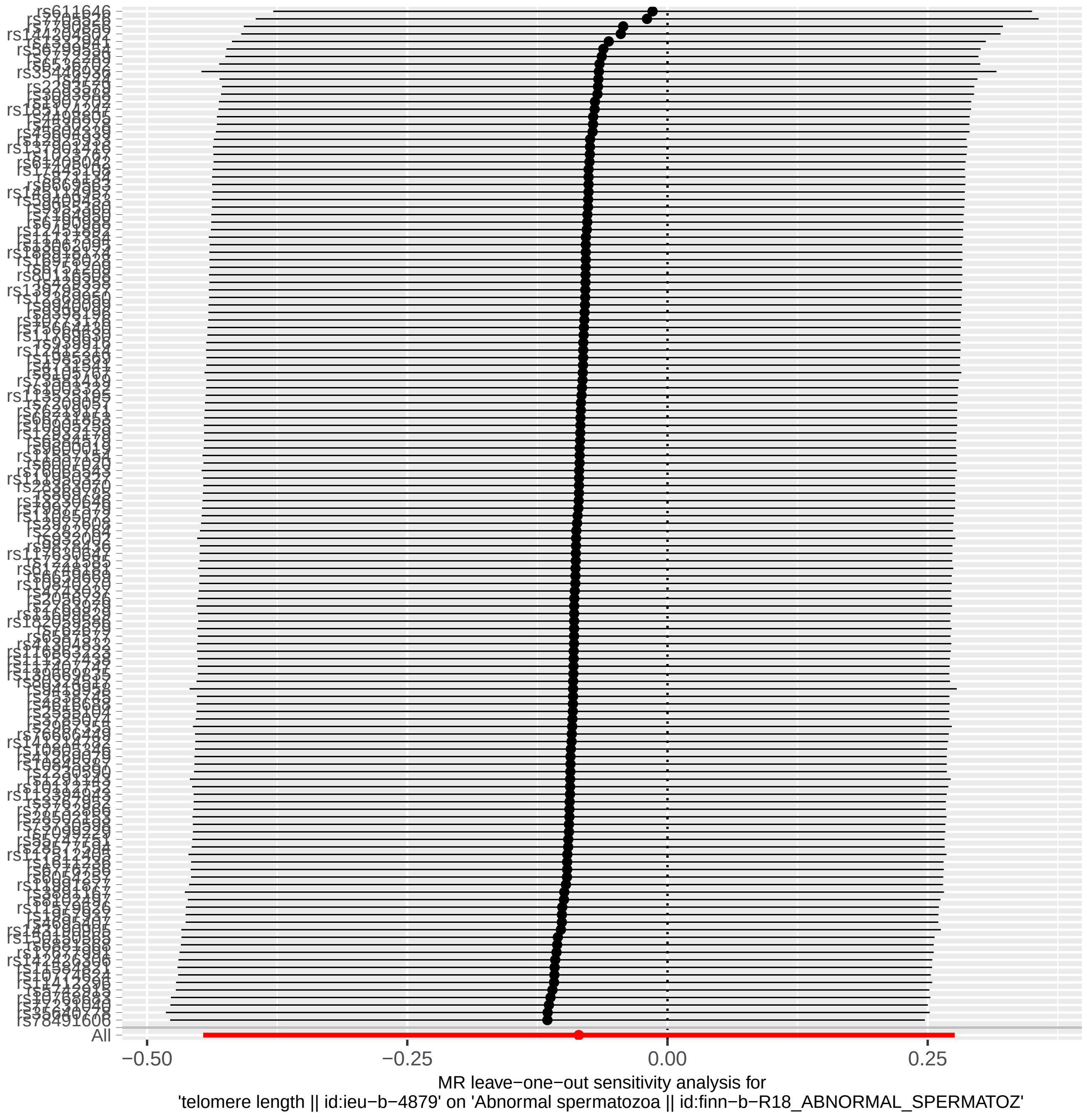
rs611646
rs78491606
All
−0.50
−0.25
0.00
0.25
MR leave−one−out sensitivity analysis for
'telomere length || id:ieu−b−4879' on 'Abnormal spermatozoa || id:finn−b−R18_ABNORMAL_SPERMATOZ'

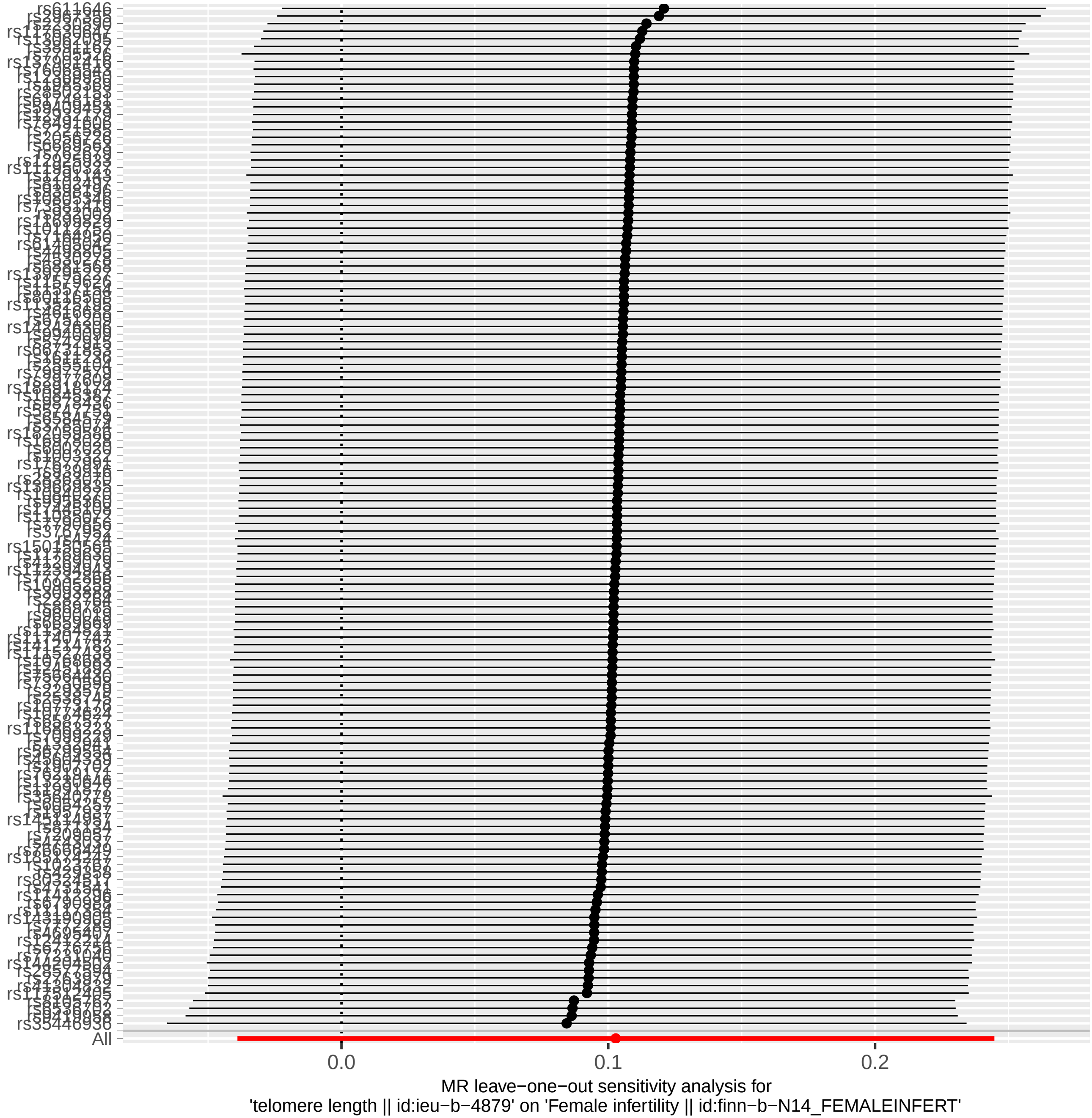
rs611646
rs35446936
All
0.0
0.1
0.2
MR leave−one−out sensitivity analysis for
'telomere length || id:ieu−b−4879' on 'Female infertility || id:finn−b−N14_FEMALEINFERT'

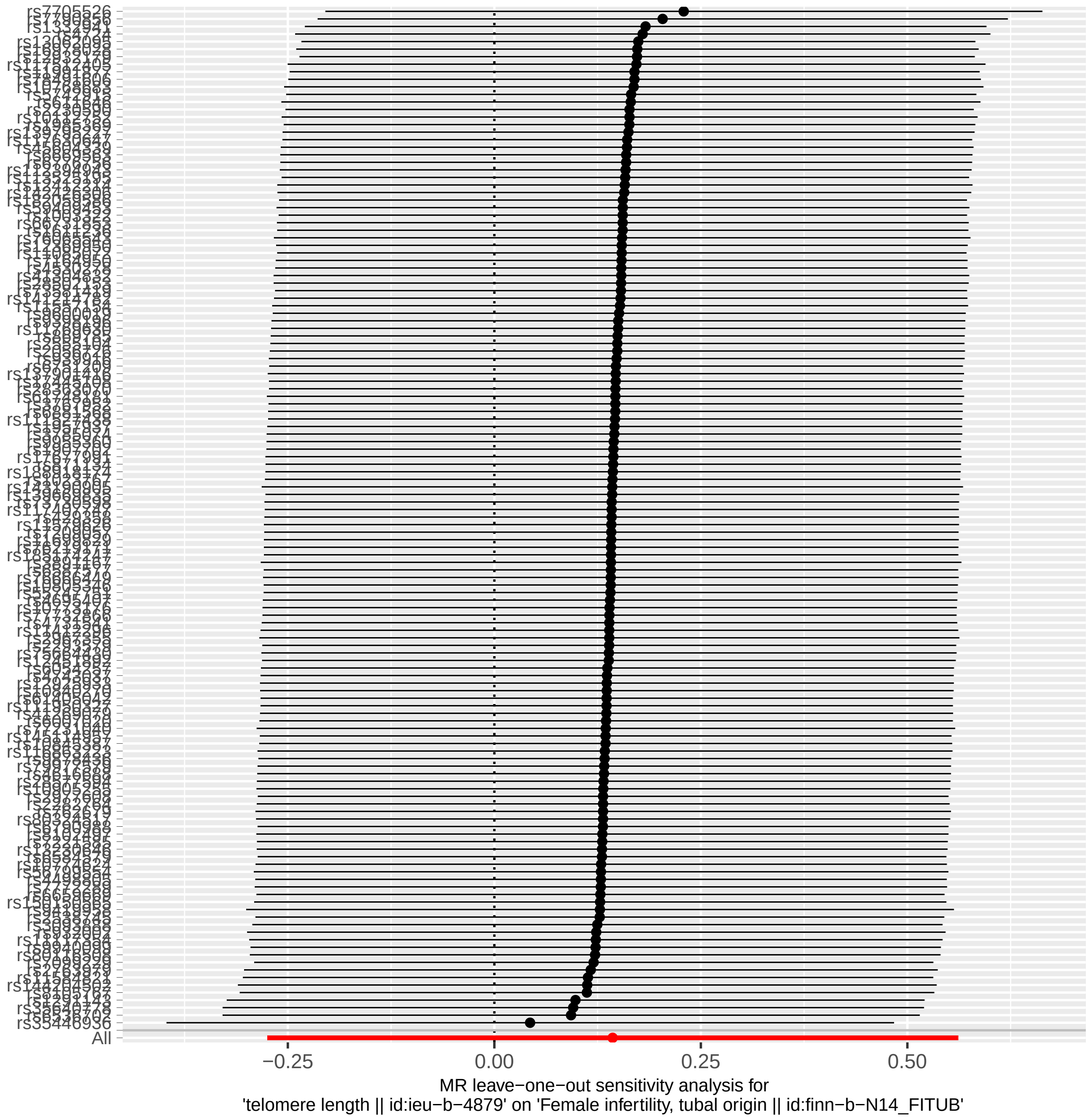

rs7705526
rs35446936
All
−0.25
0.00
0.25
0.50
MR leave−one−out sensitivity analysis for
'telomere length || id:ieu−b−4879' on 'Female infertility, tubal origin || id:finn−b−N14_FITUB'

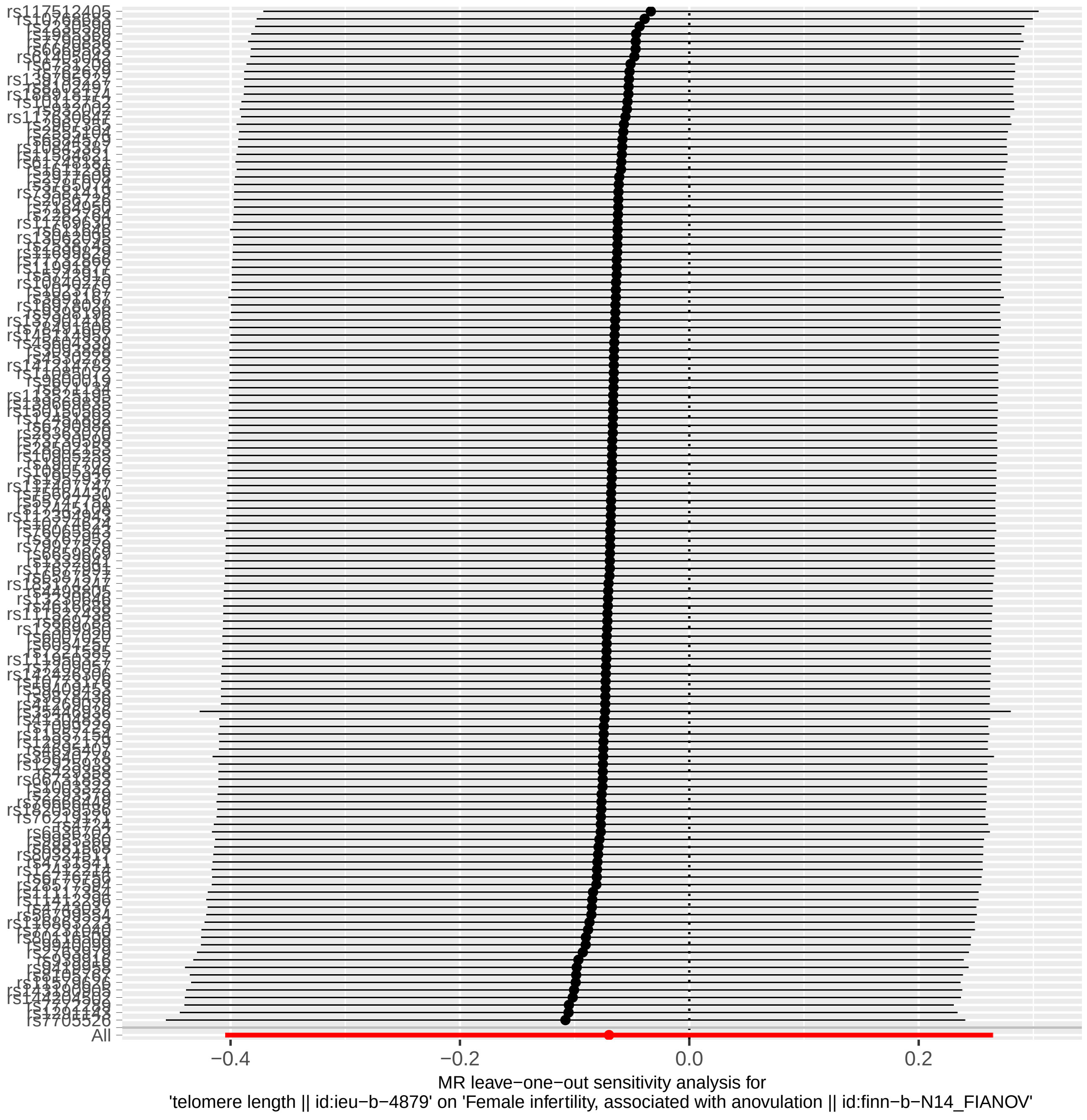
rs117512405
rs7705526
All
−0.4
−0.2
0.0
0.2
MR leave−one−out sensitivity analysis for
'telomere length || id:ieu−b−4879' on 'Female infertility, associated with anovulation || id:finn−b−N14_FIANOV'

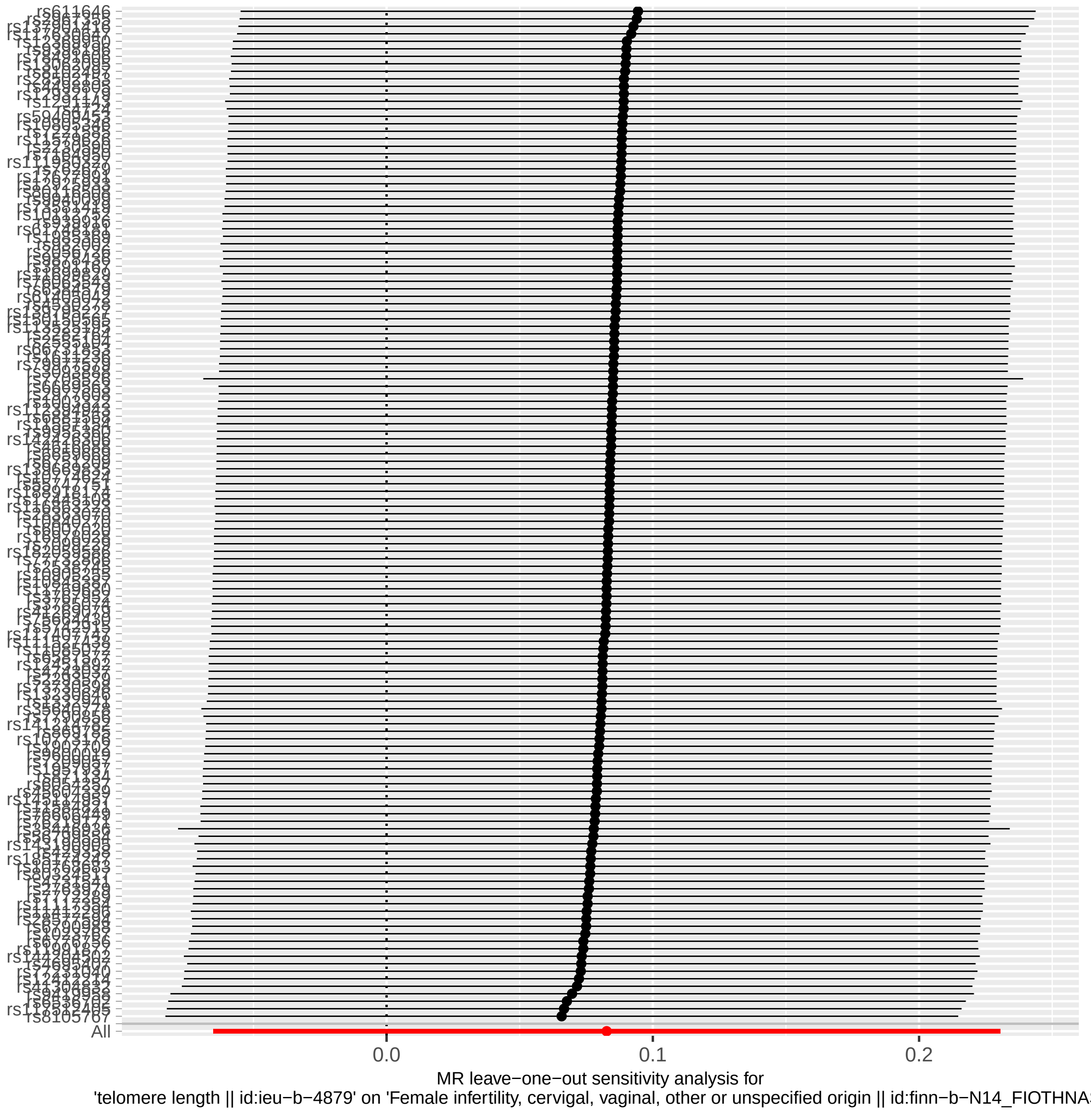
rs611646
rs8105767
All
0.0
0.1
0.2
MR leave−one−out sensitivity analysis for
'telomere length || id:ieu−b−4879' on 'Female infertility, cervigal, vaginal, other or unspecified origin || id:finn−b−N14_FIOTHNA

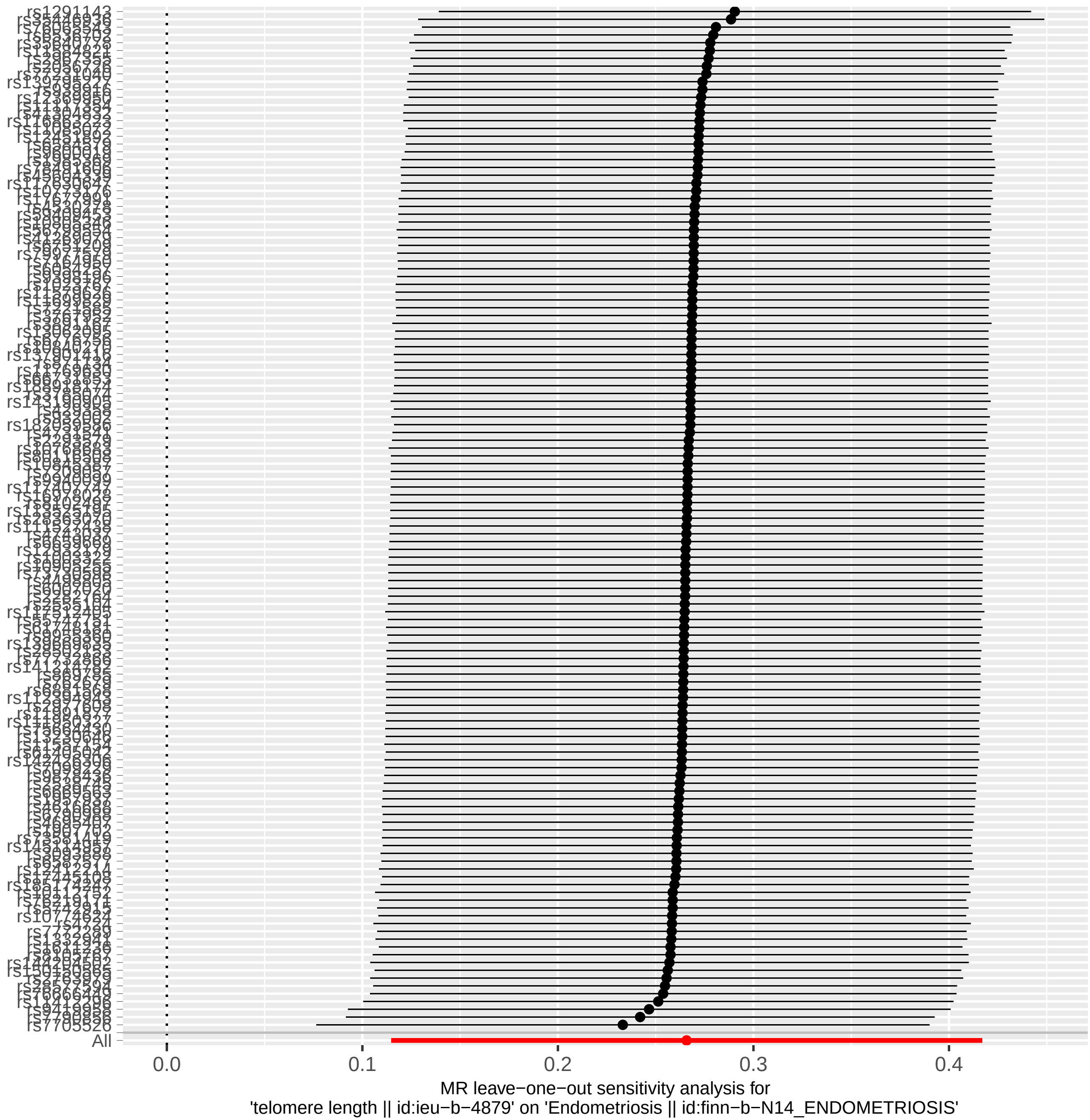
rs1291142
rs7705526
All
0.0
0.1
0.2
0.3
0.4
MR leave−one−out sensitivity analysis for
'telomere length || id:ieu−b−4879' on 'Endometriosis || id:finn−b−N14_ENDOMETRIOSIS'

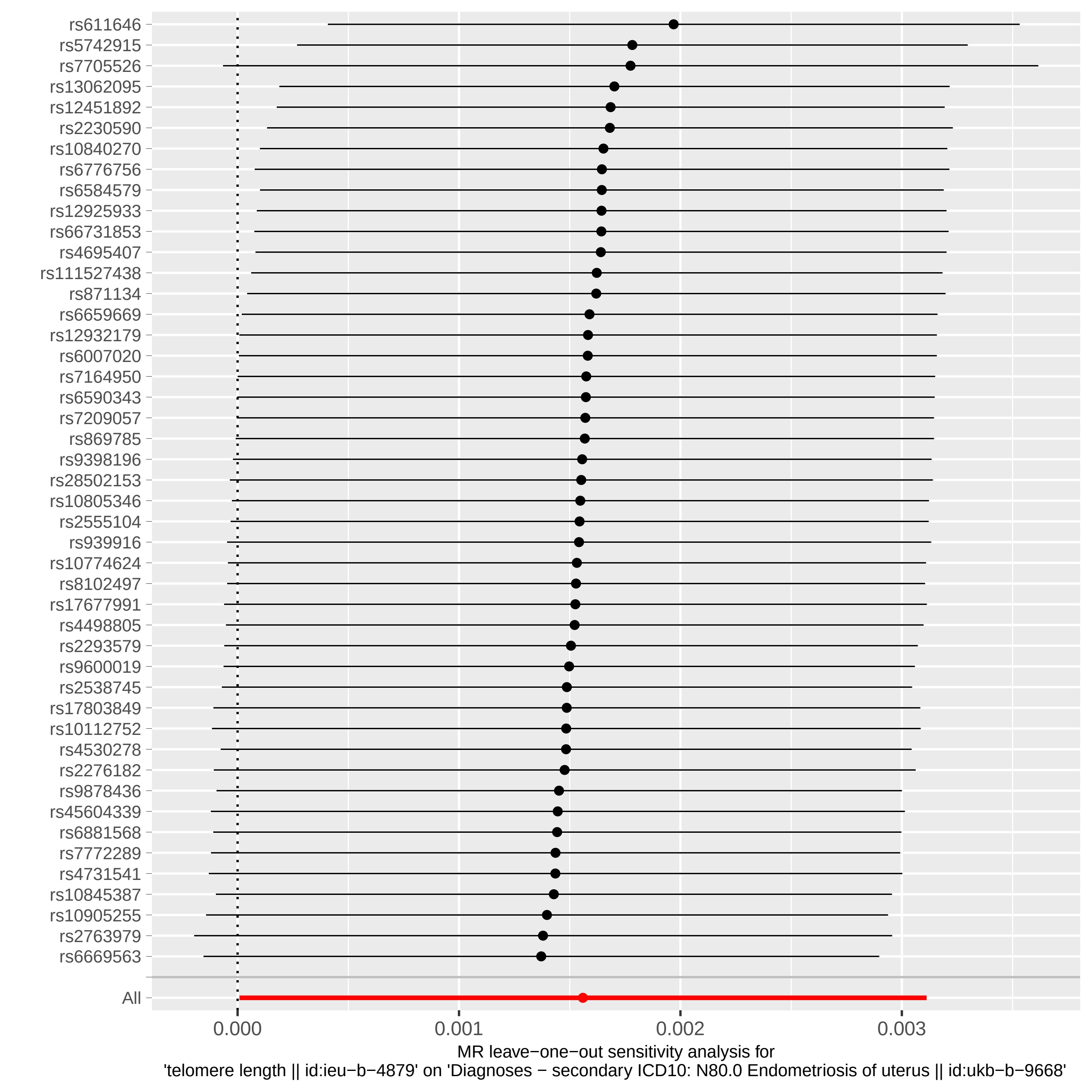
rs611646
rs5742915
rs7705526
rs13062095
rs12451892
rs2230590
rs10840270
rs6776756
rs6584579
rs12925933
rs66731853
rs4695407
rs111527438
rs871134
rs6659669
rs12932179
rs6007020
rs7164950
rs6590343
rs7209057
rs869785
rs9398196
rs28502153
rs10805346
rs2555104
rs939916
rs10774624
rs8102497
rs17677991
rs4498805
rs2293579
rs9600019
rs2538745
rs17803849
rs10112752
rs4530278
rs2276182
rs9878436
rs45604339
rs6881568
rs7772289
rs4731541
rs10845387
rs10905255
rs2763979
rs6669563
All
0.000
0.001
0.002
0.003
MR leave−one−out sensitivity analysis for
'telomere length || id:ieu−b−4879' on 'Diagnoses − secondary ICD10: N80.0 Endometriosis of uterus || id:ukb−b−9668'

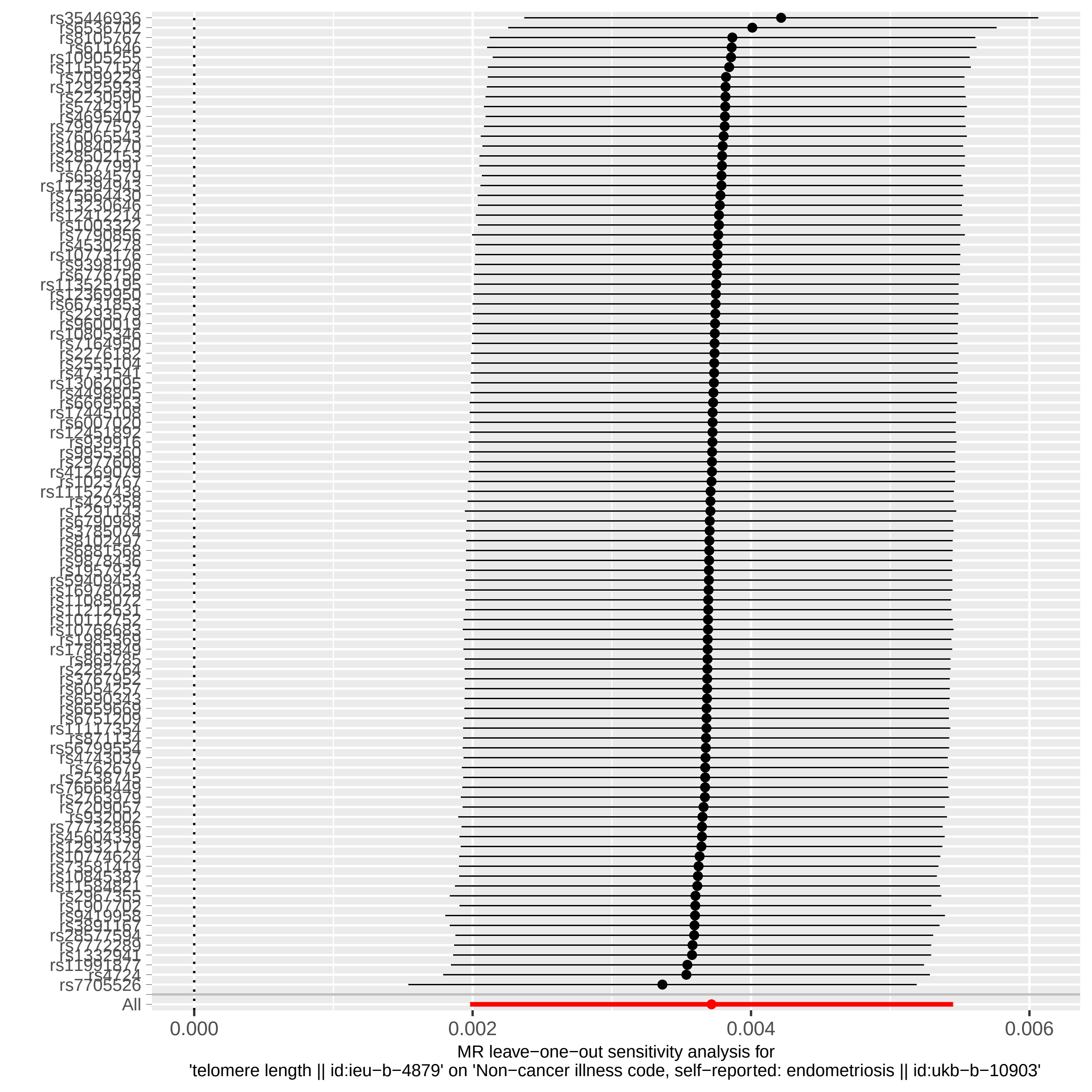
All
0.000
0.002
0.004
0.006
MR leave−one−out sensitivity analysis for
'telomere length || id:ieu-b−4879' on 'Non−cancer illness code, self−reported: endometriosis || id:ukb−b−10903'

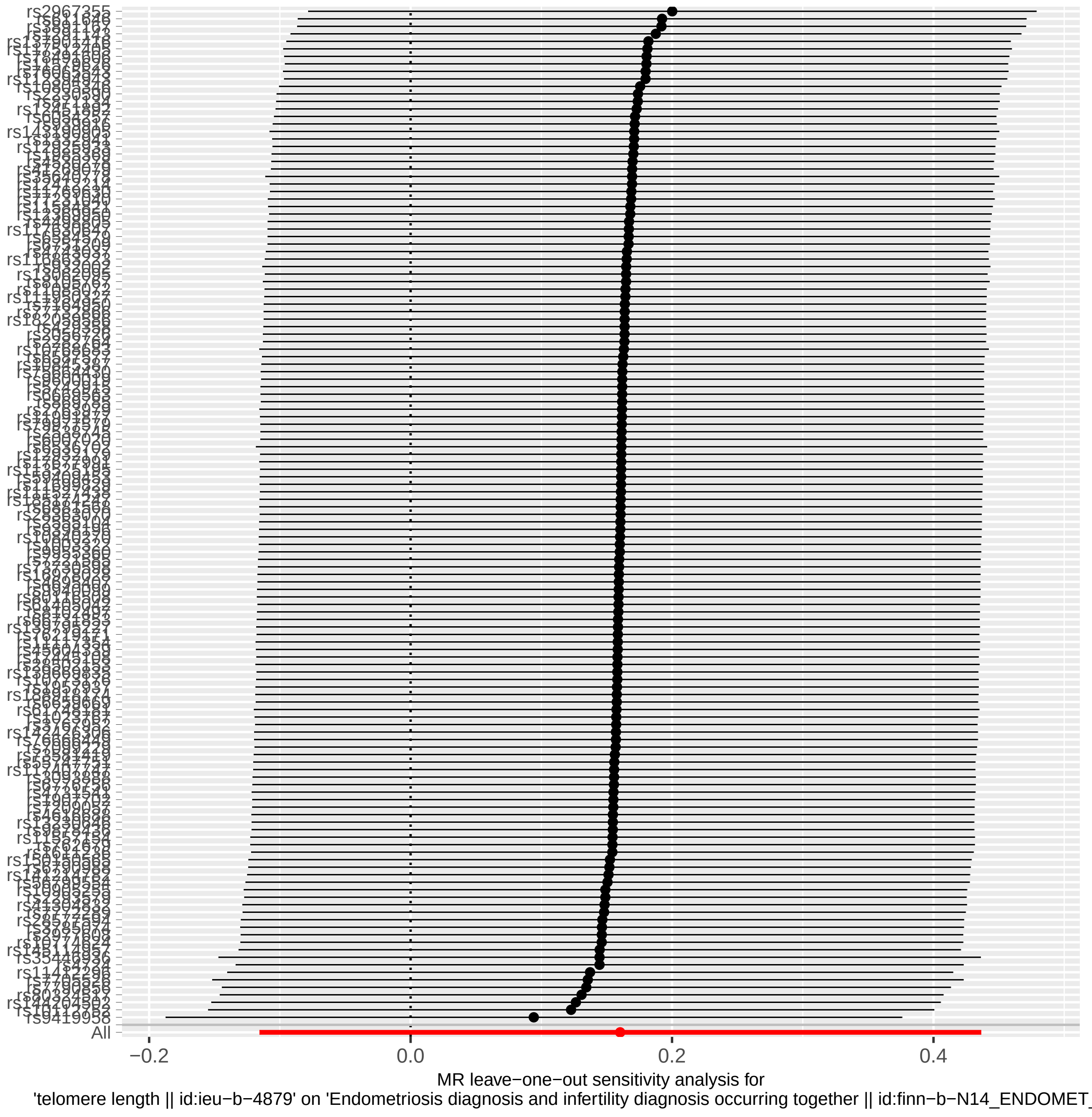

rs2967355
rs9419958
All
−0.2
0.0
0.2
0.4
MR leave−one−out sensitivity analysis for
'telomere length || id:ieu−b−4879' on 'Endometriosis diagnosis and infertility diagnosis occurring together || id:finn−b−N14_ENDOMET_

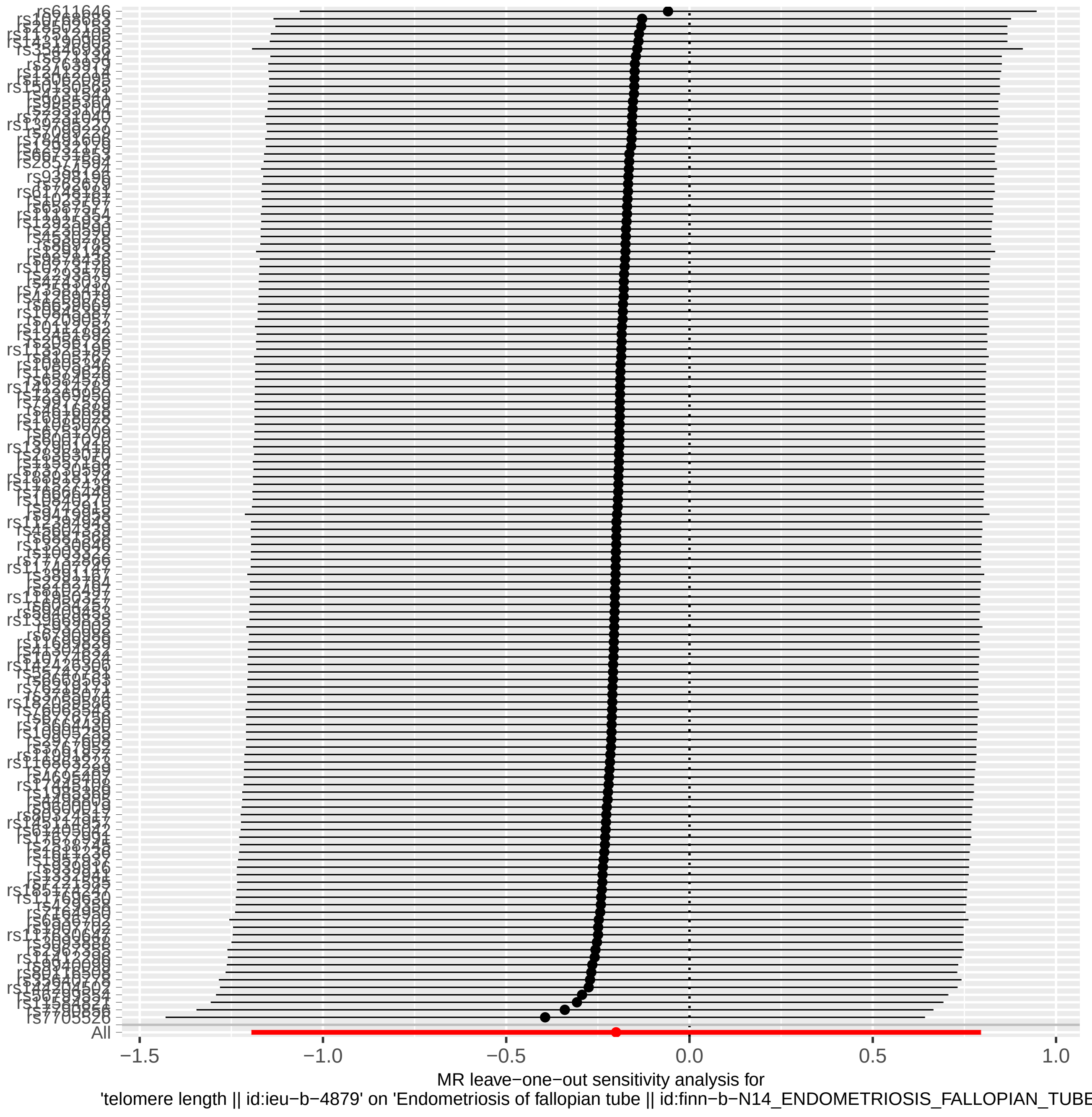
rs611646
rs7705526
All
−1.5
−1.0
−0.5
0.0
0.5
1.0
MR leave−one−out sensitivity analysis for
'telomere length || id:ieu−b−4879' on 'Endometriosis of fallopian tube || id:finn−b−N14_ENDOMETRIOSIS_FALLOPIAN_TUBE

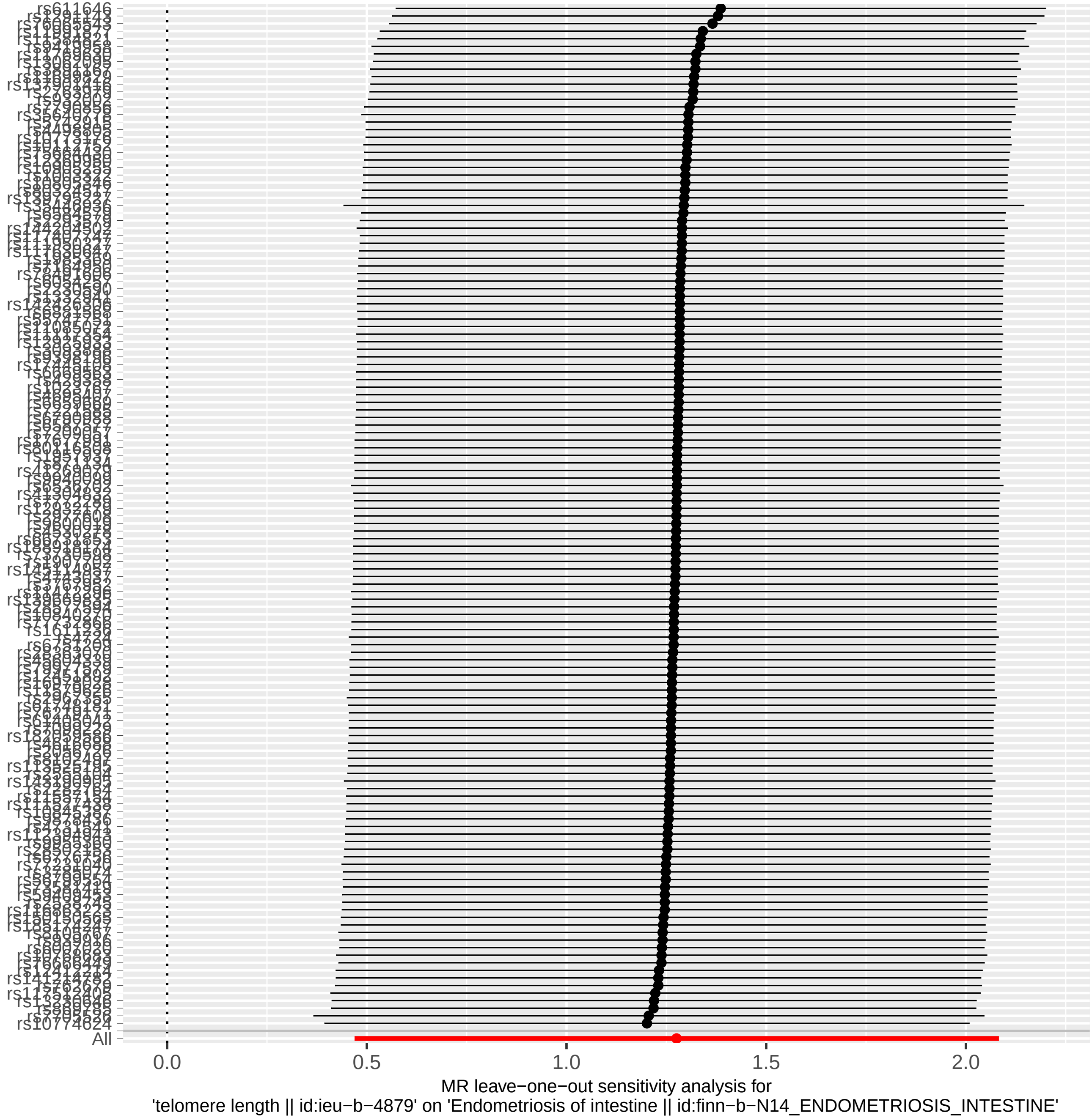
rs611646
rs10774624
All
0.0
0.5
1.0
1.5
2.0
MR leave−one−out sensitivity analysis for
'telomere length || id:ieu−b−4879' on 'Endometriosis of intestine || id:finn−b−N14_ENDOMETRIOSIS_INTESTINE'

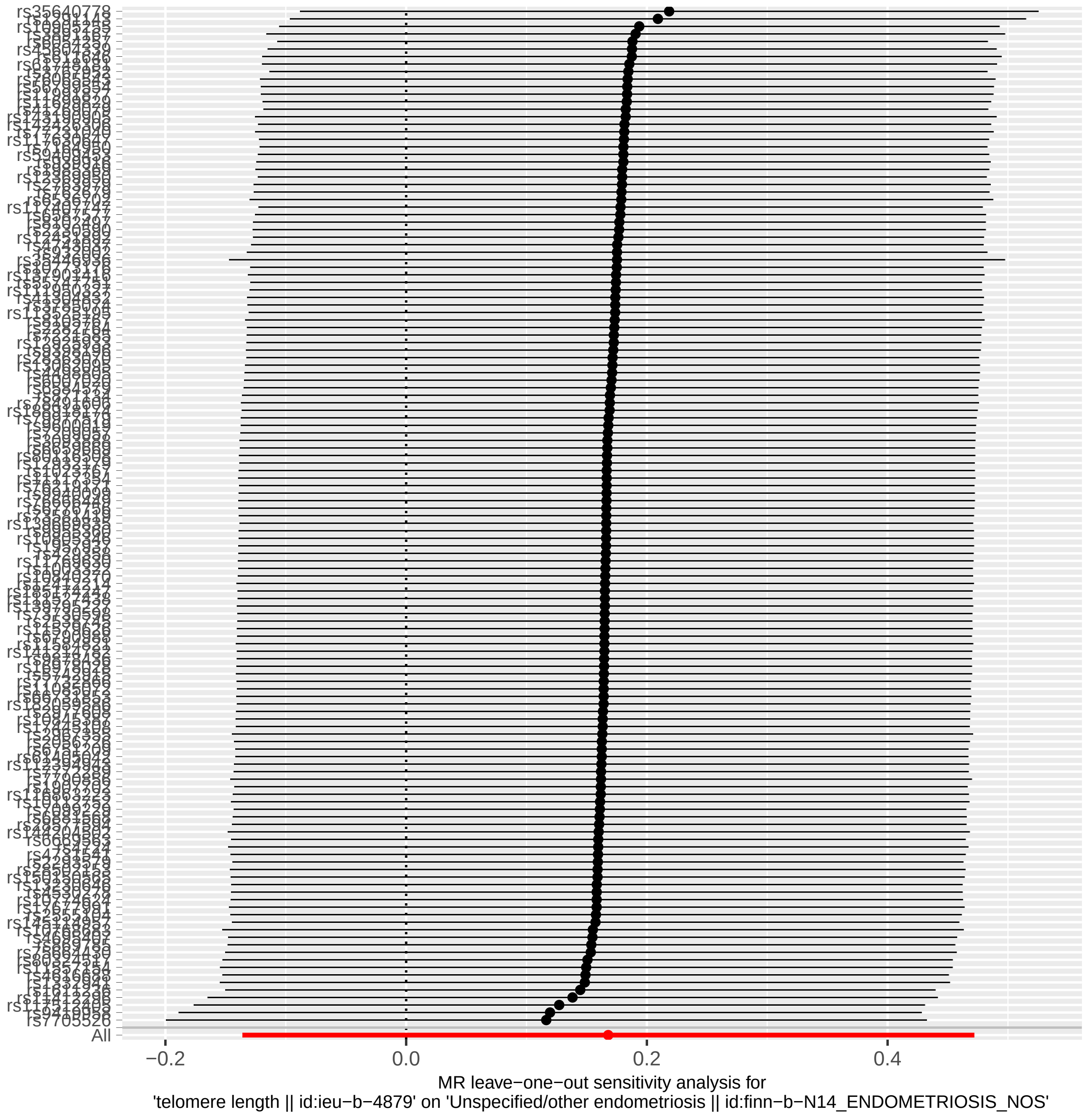
rs35640778
rs7705526
All
−0.2
0.0
0.2
0.4
MR leave−one−out sensitivity analysis for
'telomere length || id:ieu−b−4879' on 'Unspecified/other endometriosis || id:finn−b−N14_ENDOMETRIOSIS_NOS'

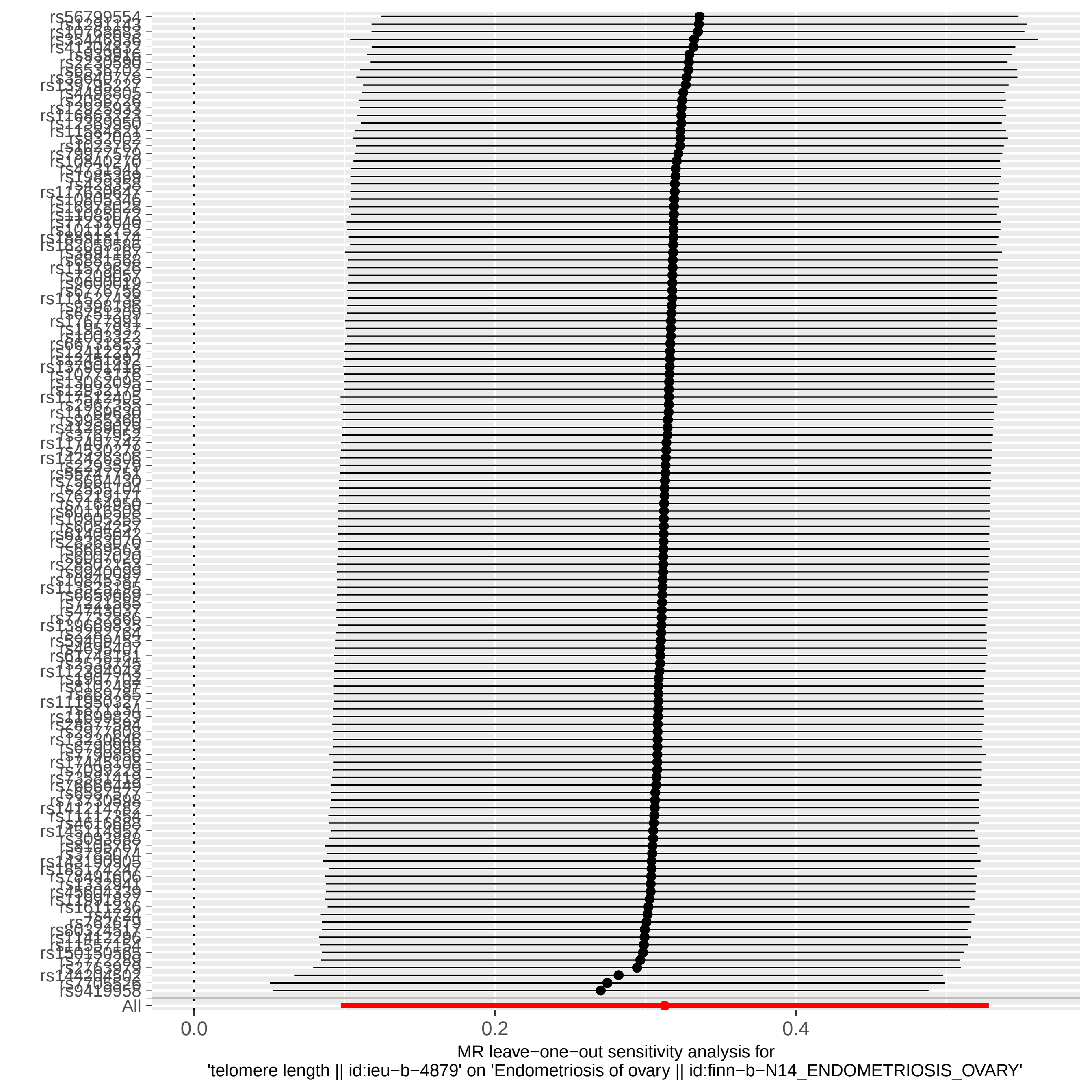
rs56799554
rs9419958
All
0.0
0.2
0.4
MR leave−one−out sensitivity analysis for
'telomere length || id:ieu−b−4879' on 'Endometriosis of ovary || id:finn−b−N14_ENDOMETRIOSIS_OVARY'

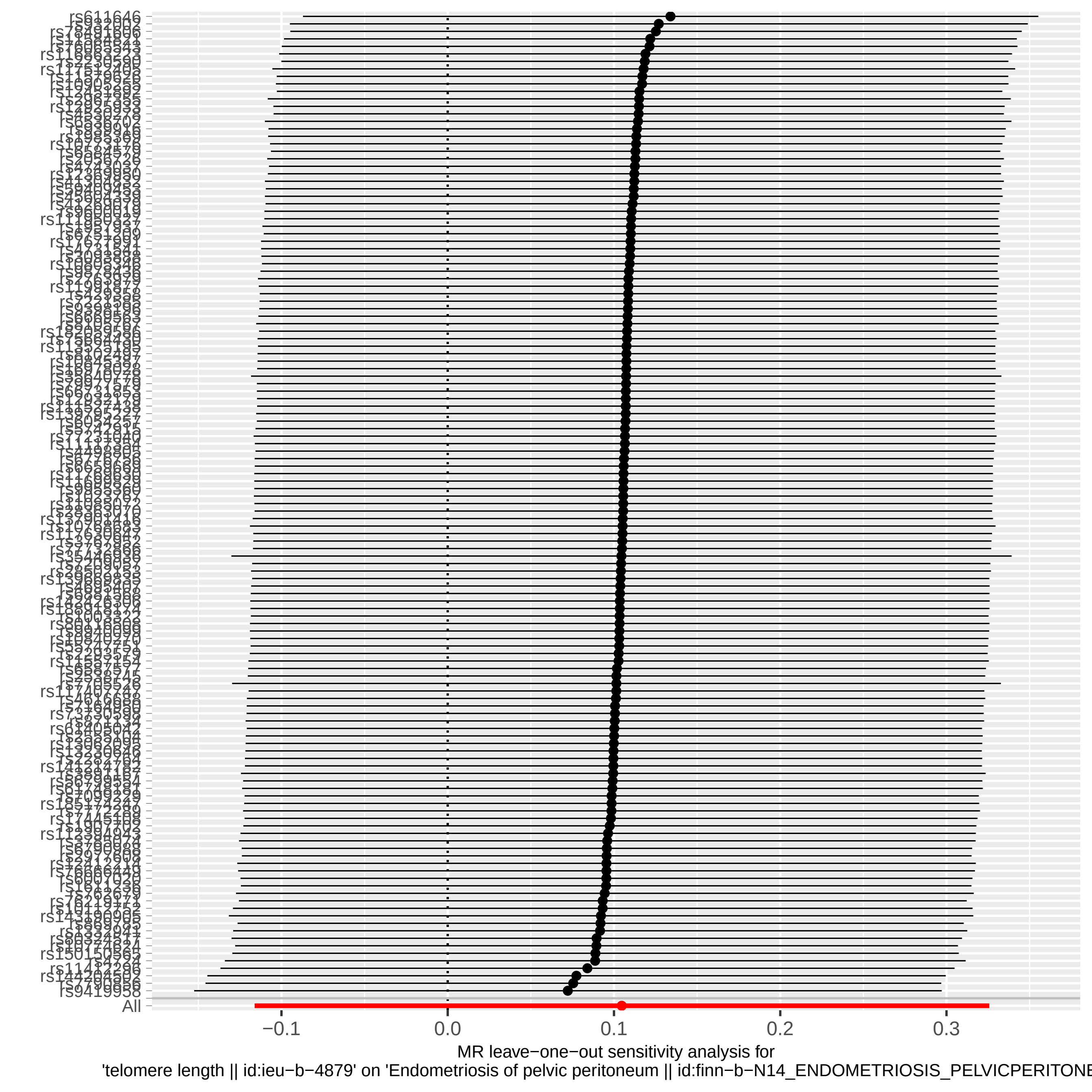
rs611646
rs9419958
All
−0.1
0.0
0.1
0.2
0.3
MR leave−one−out sensitivity analysis for
'telomere length || id:ieu−b−4879' on 'Endometriosis of pelvic peritoneum || id:finn−b−N14_ENDOMETRIOSIS_PELVICPERITONE

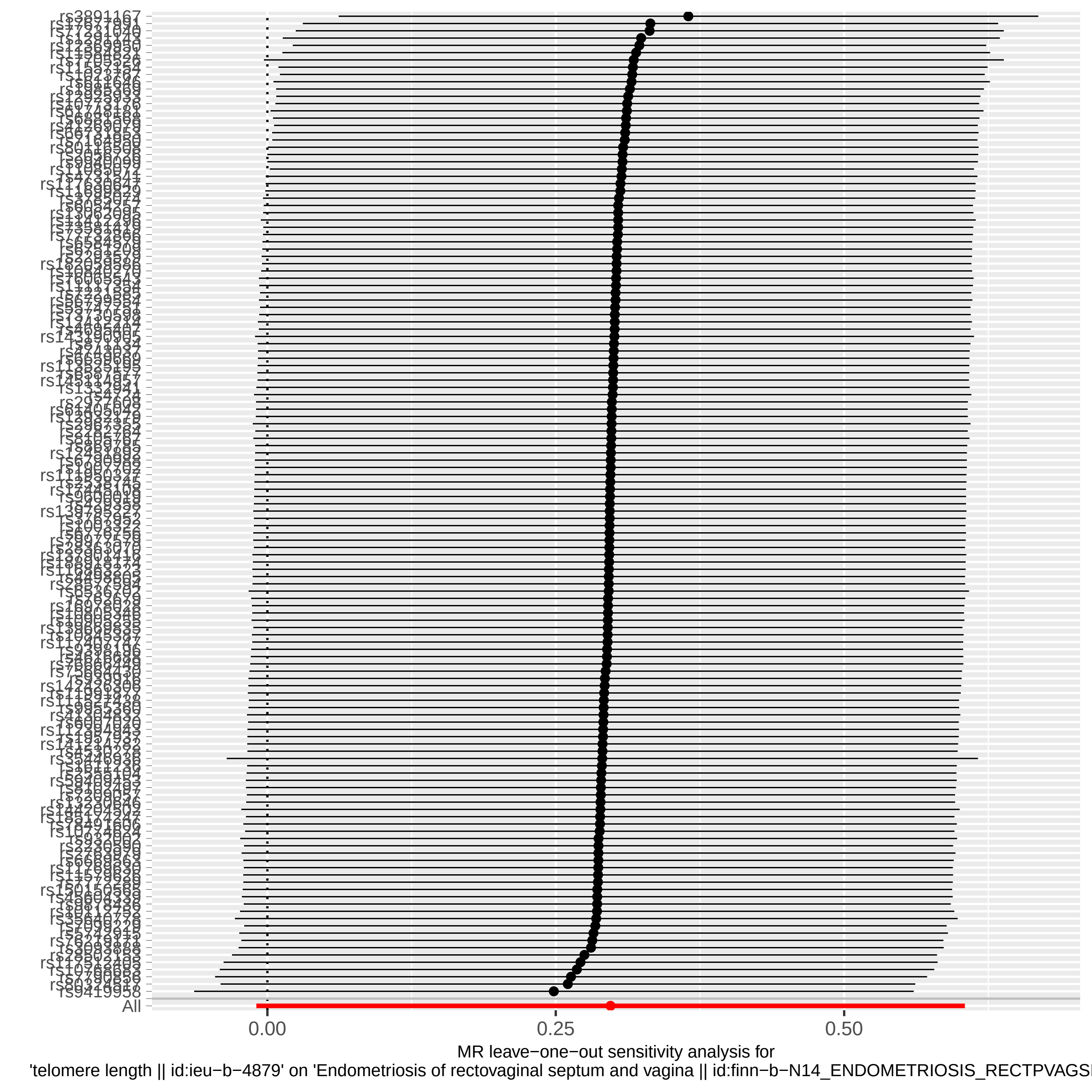
rs3891167
rs9419958
All
0.00
0.25
0.50
MR leave−one−out sensitivity analysis for
'telomere length || id:ieu−b−4879' on 'Endometriosis of rectovaginal septum and vagina || id:finn−b−N14_ENDOMETRIOSIS_RECTPVAGSEPT_VAGINA'

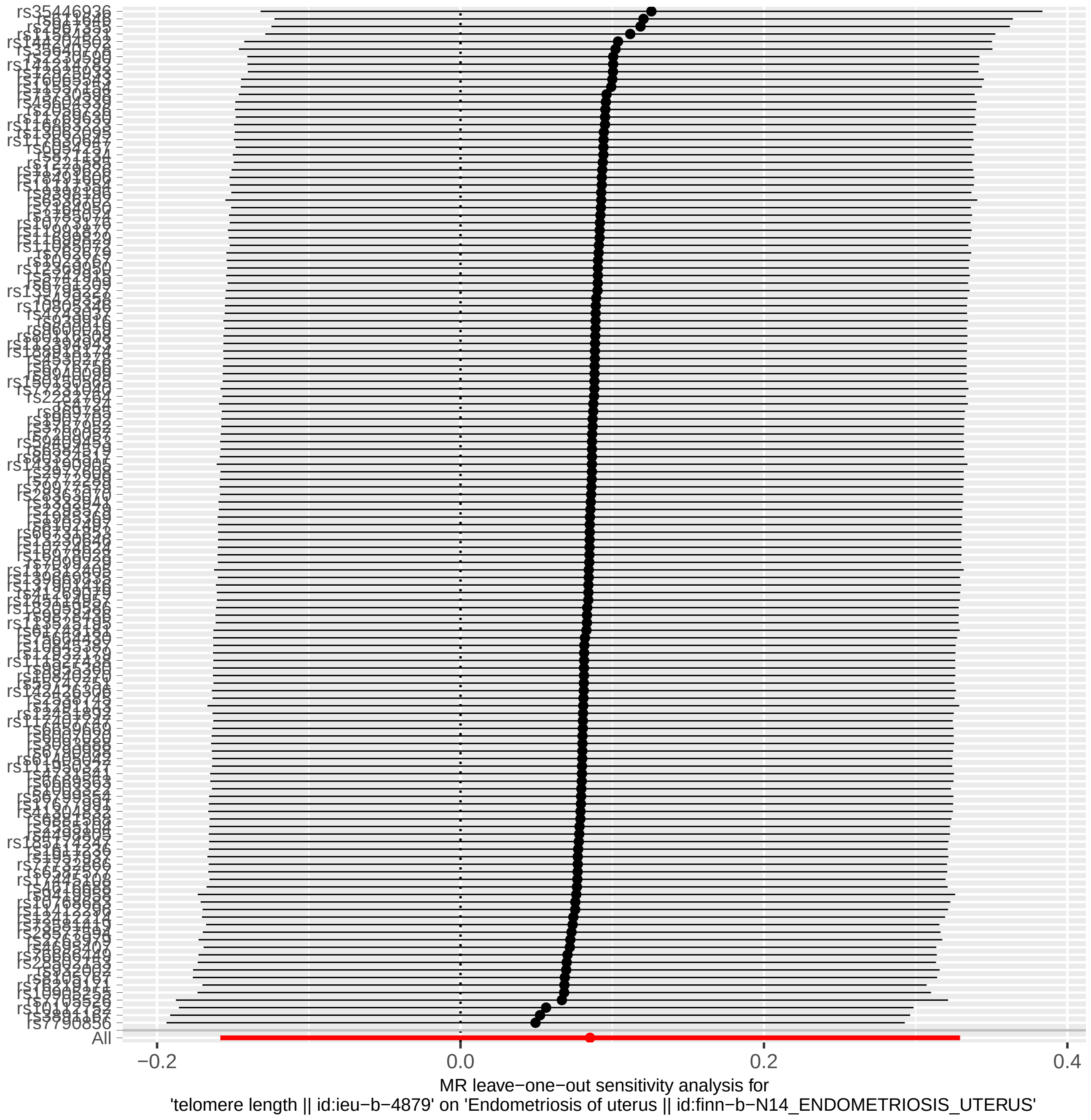
rs35446936
rs7790836
All
−0.2
0.0
0.2
0.4
MR leave−one−out sensitivity analysis for
'telomere length || id:ieu−b−4879' on 'Endometriosis of uterus || id:finn−b−N14_ENDOMETRIOSIS_UTERUS'
