# Supplementary material for: Unraveling the causal association between leukocyte telomere length and infertility: A two-sample Mendelian randomization study
Source: PLoS One. 2024 Mar 21;19(3):e0298997. doi: 10.1371/journal.pone.0298997 (PMC10956861; doi:10.1371/journal.pone.0298997)
Supplement: Attachment — Submitted filename: Response to Reviewers.docx [file pone.0298997.s009.docx]

Dear Dr. Lustig,

Thank you for the thorough review of our article and the valuable feedback provided by the reviewers. We appreciate the time and effort put into evaluating our work. We have carefully considered the revision suggestions and have made appropriate modifications to address each comment. According to the reviewers’ comments, we have tried best to modify our manuscript to meet with the requirements of your journal. In this revised version, changes to our manuscript within the document were all highlighted by using red colored text. The authors have declared that no competing interests exist. Below, we would like to respond to each point raised and provide a detailed explanation of our response to each revision suggestion.

**SUGGESTIONS FROM EDITOR**

**Editor suggestion 1:** The statistical analysis associated with MR analysis requires the confounding variates that were included in the studies. The authors need to provide clarification of this issue.

**Response 1:** We appreciate your suggestion. We have included supplementary information in the Methods section to demonstrate that the GWAS summary data of LTL have been appropriately adjusted for confounding factors, such as age and sex (refer to lines 76-77 of the revised manuscript). To address the potential confounding effects of BMI and smoking, we employed multivariable Mendelian randomization (MVMR) analysis (refer to lines 79-83 of the revised manuscript). The results of the MVMR analysis have been incorporated into Tables 1, 2, and 3, with further details provided in S7 Table.

**Editor suggestion 2:** Please upload your study’s minimal underlying data set as either Supporting Information files or to a stable, public repository and include the relevant URLs, DOIs, or accession numbers within your revised cover letter.

**Response 2**: The minimal data set we have provided in supplementary table 1. We can access the data by using URLs such as [https://gwas.mrcieu.ac.uk/datasets/ieu-b-4879/.](https://gwas.mrcieu.ac.uk/datasets/ieu-b-4877/.) ieu-b-4879 is the GWAS ID which can be found in S1 Table.

**Editor suggestion 3:** Please include captions for your Supporting Information files at the end of your manuscript, and update any in-text citations to match accordingly.

**Response 3:** We have added explanatory information about the appendix files at the end of the article. (see details at LINE376-384 of the revised manuscript)

**Editor suggestion 4:** Please review your reference list to ensure that it is complete and correct. If you have cited papers that have been retracted, please include the rationale for doing so in the manuscript text, or remove these references and replace them with relevant current references. Any changes to the reference list should be mentioned in the rebuttal letter that accompanies your revised manuscript. If you need to cite a retracted article, indicate the article’s retracted status in the References list and also include a citation and full reference for the retraction notice.

**Response 4:** There wasn’t retracted articles in our reference. In the revised manuscript, we added a new reference article for smoking GWAS summary data (“Association studies of up to 1.2 million individuals yield new insights into the genetic etiology of tobacco and alcohol use”, new Ref #11). According to reviewers’ advice, we also deleted reference articles “Ageing and telomeres: a study into organ and gender specific telomere shortening” (Raw Ref #21) and “Oxidative stress induces persistent telomeric DNA damage responsible for nuclear morphology change in mammalian cells” (Raw Ref #22) in discussion parts.

**COMMENTS TO THE AUTHOR**

**MAJOR COMMENTS**

**Reviewer #1:** Did the authors perform adjustment for any important covariates in their analyses such as age and sex? Although MR operates under the assumption that instrumental variables do not act on confounders, it does not preclude the existence of those confounders. Important confounds of interest (age, sex, study site, BMI) should be included in the first stage regression to generate IVs for SNP-LTL and SNP-infertility related traits or as covariates in the second stage regression. For further details see Statistical Analysis of Kuo et al., 2019 (https://doi.org/10.1111/acel.13017) and Discussion of Burgess et al., 2011; <https://doi.org/10.1093/ije/dyr036).>

**Response 1:** Thanks for your valuable advice. We have thoroughly reviewed the recommended articles, specifically focusing on the statistical analysis conducted by Kuo. In their study, he used SNPs on mean LTL was previously estimated with adjustment for age, sex, body mass index (BMI), and smoking history (Haycock et al., 2017; <https://doi.org/10.1001/jamaoncol.2016.5945).> However, we were unable to obtain the same GWAS summary data used in their analysis. In our study, the summary GWAS data of LTL was adjusted for age, sex, array, and the first ten principal components. To account for the potential confounding effects of BMI and smoking history, we employed multivariable Mendelian randomization (MVMR) methods. The MVMR results have been included in Tables1-Table3, and further detailed information can be found in S7 Table. In summary, even after excluding the effects of confounding factors such as BMI and smoking history, we did not observe a significant causal relationship between LTL and infertility, while a statistically significant association between LTL and endometriosis was still observed. As suggested, we have provided a detailed description in the Exposure data section of the Methods and highlighted the changes in yellow.

**Modified (LINE 75-83):** The genetic association data for LTL were obtained from a genome-wide association study (GWAS) involving 472,174 European participants (10), adjusting for age, sex, and the first ten principal components (PCs). These participants were aged 40-69 years and had an equal distribution of males (45.8%) and females (54.2%). LTL measurements were determined using a quantitative polymerase chain reaction (qPCR) assay. To mitigate any potential biases stemming from body mass index (BMI) and smoking, we procured comprehensive BMI GWAS summary data from the UK Biobank and smoking GWAS sunmmary data from the GWAS & Sequencing Consortium of Alcohol and Nicotine use (GSCAN). Leveraging these datasets, we conducted multivariable Mendelian Randomization (MVMR) to ensure a robust analysis.

**Modified Table 1-3:**

Table 1 Results of MR analysis between LTL and male infertility

| **Outcomes** | **OR** | **95%CI** | **p** | **p_pleiotropy_** | **p_heterogeneity_** | **p_mr-presso_** | **p_MVMR_** |
| --- | --- | --- | --- | --- | --- | --- | --- |
| Male infertility | 1.269 | (0.838,1.921) | 0.261 | 0.825 | 0.97 | 0.974 | 0.286 |
| Abnormal spermatozoa | 0.918 | (0.640,1.317) | 0.643 | 0.842 | 0.771 | 0.781 | 0.381 |

Table 2 Results of MR analysis between LTL and female infertility

| **Outcomes** | **OR** | **95%CI** | **p** | **p_pleiotropy_** | **p_heterogeneity_** | **p_mr-presso_** | **p_MVMR_** |
| --- | --- | --- | --- | --- | --- | --- | --- |
| Female infertility | 1.108 | (0.962,1.277) | 0.155 | 0.199 | 0.547 | 0.557 | 0.132 |
| Female infertility, associated with anovulation | 0.932 | (0.667,1.303) | 0.682 | 0.159 | 0.623 | 0.630 | 0.679 |
| Female infertility, cervigal, vaginal, other or unspecified origin | 1.086 | (0.937,1.259) | 0.273 | 0.164 | 0.647 | 0.667 | 0.175 |
| Female infertility, tubal origin | 1.154 | (0.760,1.753) | 0.502 | 0.774 | 0.100 | 0.086 | 0.645 |
| Endometriosis diagnosis and infertility diagnosis occurring together | 1.174 | (0.891,1.547) | 0.255 | 0.396 | 0.536 | 0.529 | 0.640 |

Table 3 Results of MR analysis between LTL and endometriosis

| **Outcomes** | **Consortiumm** | **OR** | **95%CI** | **p** | **p_pleiotropy_** | **p_heterogeneity_** | **p_mr-presso_** | **p_MVMR_** |
| --- | --- | --- | --- | --- | --- | --- | --- | --- |
| Endometriosis | FinnGen | 1.304 | (1.122,1.517) | 0.001 | 0.896 | 0.023 | 0.019 | 0.023 |
| ICD10: N80.0 Endometriosis of uterus | UKbiobank | 1.002 | (1.000,1.003) | 0.049 | 0.912 | 0.269 | 0.270 | 0.124 |
| Self-reported: endometriosis | UKbiobank | 1.004 | (1.002,1.005) | 2.65E-05 | 0.870 | 0.164 | 0.155 | 0.002 |

**Reviewer #2:** Although the authors provide a supplementary table with a full list of SNPs utilized to generate IVs for LTL and infertility related traits, it would be beneficial to also include a summary table of the total number of SNPs associated with each factor and how much variance in each factor was explained by the overall instrument.

**Response 2:** Thank you for your valuable suggestions. We acknowledge the significance of providing a comprehensive summary of the single nucleotide polymorphisms (SNPs) utilized as instrumental variables (IVs) for both telomere length (LTL) and infertility-related traits. To address this, we have included a summary table, namely S3 Table, in our revised manuscript. This table presents detailed information regarding the total number of SNPs associated with each factor, as well as the mean F-statistics and the sum of R2 values. In our study, we utilized a total of 135 instrumental variables. The mean F-statistics for these instrumental variables is approximately 120, indicating strong instrument strength. Furthermore, the sum of R2 values for the SNPs is approximately 0.01, suggesting that the SNPs explain a small proportion of the variance in the traits of interest.

Added S3 Table:

**MINOR COMMENTS**

**Reviewer #3:** It should be made clear that the data used for analyses of male and female infertility was derived from the FinnGen datasets in a similar manner as is described for the analyses on LTL and endometriosis.

**Response 3:** Thanks for your valuable advice. We have added the data description in the results parts of male and female infertility.

**Added (LINE 131-133):** We conducted a comprehensive analysis using the male infertility and sperm abnormalities datasets from FinnGen to evaluate the causal association between leukocyte telomere length (LTL) and the occurrence of male infertility and sperm abnormalities.

**Added (LINE 142-144):** To investigate the potential causal relationship between leukocyte telomere length (LTL) and female infertility, we conducted a rigorous analysis utilizing the comprehensive collection of five datasets obtained from FinnGen.

**Reviewer #4:** It would help to contextualize contrasting results between TL and male infertility and their possible relationship to sample size by including the sample size of Ref #7 to contrast it with the “larger study” in Ref #20. That said, it is unclear if sample size contributes to the discrepancy of findings at all since the study on nonobstructive azoospermia (Ref #19) had a sample size of 866. Instead it might be a difference of outcome and how male infertility is defined.

**Response 4:** We think this is an excellent suggestion. Upon further examination of the relevant literature, it was discovered that Reference #19 also supports the absence of a correlation between telomere length (LTL) and obstructive azoospermia (OA), while indicating a correlation between LTL and nonobstructive azoospermia (NOA). OA and NOA are two subtypes of oligozoospermia. Both Reference #7 and Reference #20 conducted research on male infertility populations, but they did not analyze the specific subtypes of oligozoospermia as explored in Reference #19. Reference #7 did not account for confounding factors such as age in their statistical analysis, whereas Reference #20 had a substantial sample size and effectively controlled for confounding factors including age and smoking, rendering its findings more reliable. Considering these factors, we have revised the discussion section regarding the association between LTL and male infertility.

**Revised paragraph (LINE 200-209):** Nevertheless, upon meticulous exclusion of the confounding influences of age, BMI, and smoking, our study findings robustly challenge the prevailing belief that a causal link exists between LTL and male infertility or abnormal spermatogenesis. Importantly, these results align with a comprehensive investigation encompassing 599 individuals [21], lending further credibility to our conclusion. The discrepancy in findings between our study and Yang's study [20] may be attributed to differences in the definition of sperm abnormalities. Specifically, Yang’s study found a significant relationship between LTL and NOA, whereas other types of sperm abnormalities were not significant. Furthermore, it is important to note that Darmishonnejad's study [7] did not account for the confounding effects of age, BMI, and smoking, and had a relatively small sample size, which increases the likelihood of false positive results.

Once again, we would like to express our gratitude for your and the reviewers' valuable feedback on our study. We highly value your suggestions and have taken them seriously, making the necessary revisions to our academic article. We believe these modifications will further enhance the quality and credibility of our research. If you have any further suggestions or comments on our revisions, please feel free to let us know. We look forward to your re-evaluation of our revised article.

Thank you for your time and attention.

Sincerely,

Chenghui Zhao
